# Light-Triggered Soft Artificial Muscles: Molecular-Level Amplification of Actuation Control Signals

**DOI:** 10.1038/s41598-017-08777-2

**Published:** 2017-08-23

**Authors:** Michael P. M. Dicker, Anna B. Baker, Robert J. Iredale, Sina Naficy, Ian P. Bond, Charl F. J. Faul, Jonathan M. Rossiter, Geoffrey M. Spinks, Paul M. Weaver

**Affiliations:** 1 Bristol Composites Institute (ACCIS), Queen’s School of Engineering, University of Bristol, Bristol, BS8 1TR UK; 2 School of Chemistry, University of Bristol, Bristol, BS8 1TS UK; 3 Intelligent Polymer Research Institute, ARC Centre of Excellence for Electromaterials Science, University of Wollongong, Wollongong, NSW 2522 Australia; 4 School of Chemical and Biomolecular Engineering, The University of Sydney, Sydney, NSW 2006 Australia; 5 Department of Engineering Mathematics, Merchant Venturers School of Engineering, University of Bristol, Bristol, BS8 1UB UK; 6 Bristol Robotics Laboratory, Bristol, BS34 8QZ UK

## Abstract

The principle of control signal amplification is found in all actuation systems, from engineered devices through to the operation of biological muscles. However, current engineering approaches require the use of hard and bulky external switches or valves, incompatible with both the properties of emerging soft artificial muscle technology and those of the bioinspired robotic systems they enable. To address this deficiency a biomimetic molecular-level approach is developed that employs light, with its excellent spatial and temporal control properties, to actuate soft, pH-responsive hydrogel artificial muscles. Although this actuation is triggered by light, it is largely powered by the resulting excitation and runaway chemical reaction of a light-sensitive acid autocatalytic solution in which the actuator is immersed. This process produces actuation strains of up to 45% and a three-fold chemical amplification of the controlling light-trigger, realising a new strategy for the creation of highly functional soft actuating systems.

## Introduction

**Figure 1:**
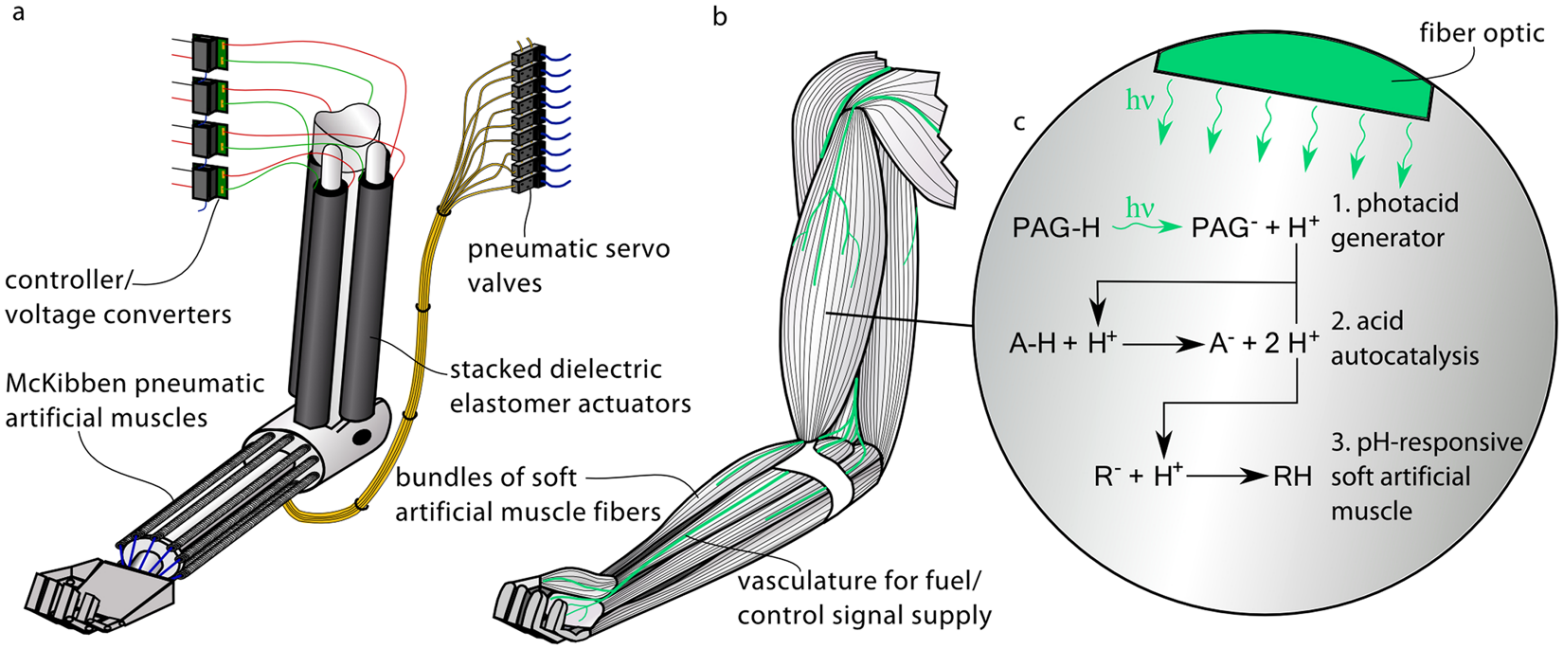
Comparison between current artificial-muscle-based robotic systems and the proposed bioinspired systems enabled by molecular-level control signal amplification. (**a**) Conventional robotic system. While the use of multiple soft artificial muscles for robotic actuation can lead to both a large range of motion and high performance (specific power, safety, selective recruitment, redundancy, morphological computation etc.), such an arrangement requires the use of many hard and bulky external switches or valves, incompatible with both the properties of the soft artificial muscles and those of future bioinspired robotic systems. (**b**) Proposed molecular-level-controlled robotic system. The deficiency of current systems is addressed in this study through the development of a molecular-level approach to control signal amplification. (**c**) Detail of investigated system: (1) light generates an acid, (2) which triggers an acid autocatalytic reaction, (3) resulting in the contraction of a pH-responsive soft artificial muscle. Such an approach enables the development of biomimetic robots containing thousands of individually addressable soft artificial muscles, controlled with the smallest of inputs transmitted through embedded fibre optics and the individual artificial muscle fibres.

The amplification of low-energy control signals into a larger supply of actuation energy is key to the precise yet powerful operation of all movement systems. The process allows for efficient implementation of control strategies and the delivery of control signals without simultaneously imposing limits on the amount of power that can be supplied to drive the motion. As a result, it is a system property found everywhere from hydraulic actuators, controlled by electric servo valves yet actuated by pressurized fluids^1, 2^, to electric motors that use driver circuits to amplify control signals^3, 4^. The most refined example of this property is found in the operation of skeletal muscles. In humans there are over 600 anatomically distinct muscles, in many cases composed of hundreds of thousands of muscle fibres, each 10–100 μm in diameter^5^. Individual groups, of as little as two and up to several thousand muscle fibres, are addressed by separate motor neurons, leading to an extreme resolution of control signalling^5–7^. This large array of control variables allows for a broad range of sophisticated movements, and a level of dexterity far beyond that of any machine^5^. Achieving such an extensive control arrangement, within a sufficiently small volume to facilitate movement, is only possible due to the molecular-level ability of low energy neural impulses (control signals) to trigger high power actuation. While the muscle contraction is triggered by this neural signal, it is powered by chemical energy stored and converted locally in the muscles and replenished through the cardiovascular system. As depicted in Fig. 1, the development of such a molecular-level approach for control signal amplification is needed for the next iteration of bioinspired robots^8–11^.

One element required for the development and application of such biomimetic machines is artificial muscles. Artificial muscles are actuator materials or ‘solid-state’ actuator devices which to some extent mimic the function and form of skeletal muscle. Artificial muscles are now available that match or exceed the mechanical performance of skeletal muscle, while also having muscle-like properties of low stiffness (compliance) and the ability to pack tightly into small areas^12–14^. However, there has been no development of artificial muscle systems that allow for biologically inspired energy and control architectures, where control signal amplification takes place at the molecular-level. This work addresses this deficiency by embracing a strategy of open systems – a common principle in the natural world^15, 16^ – to keep a light-sensitive autocatalytic solution far from thermodynamic equilibrium. Upon a short light pulse the system is sufficiently disturbed to drive the autocatalytic reaction quickly to equilibrium, molecularly amplifying the light-trigger and actuating a soft chemically responsive artificial muscle in the process. The use of autocatalysis in this way, beyond common combustion^17, 18^, has not been employed for actuation previously. Development of such light-triggered autocatalytic systems is expected to provide a route to the development of actuation systems that combine the benefits of the spatial and temporal control offered by light^19^, with the high energy density of chemical fuels.

As a first demonstration of this approach we employ a commercially available photoacid generator (PAG), 2-nitrobenzaldehyde (NBA), which rearranges and dissociates as per Reaction 1 into an acid (H^+^) upon exposure to light^20–22^. This acid triggers the autocatalytic Landolt (iodate−sulphite) version of the iodine clock reaction^23^, a popular classroom science demonstration and described qualitatively by Reaction 2 and 3^24, 25^. Here the autocatalytic role of both acid and iodide (I^−^) is observed, as they are both required for the reaction to proceed. However, these components are not consumed in the process, but are, in fact, generated in excess. While these catalytic components will be produced by the slow oxidation of bisulphite (HSO_3_^−^) by iodate (IO_3_^−^) (as shown in Reaction 2), Reaction 3 becomes dominant upon illuminated acid generation (or after sufficient time). Here iodide and acid facilitate the reduction of iodate into iodine (I_2_), leading to the rapid oxidation of bisulphite and generation of evermore iodide and acid (Reaction 3). The more acid and iodide that is produced or added, the faster this reaction takes place, with the reaction proceeding in a chain-like fashion quickly to equilibrium.1


**Figure 2:**
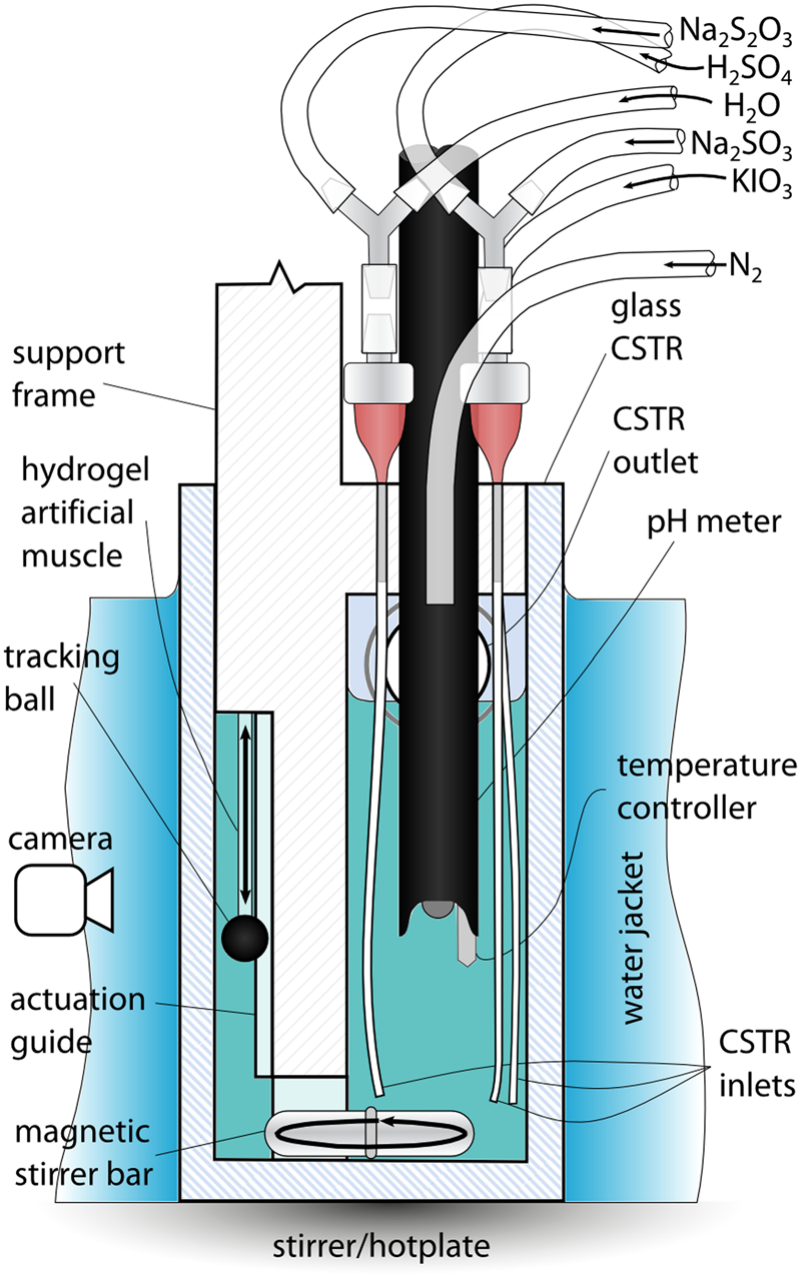
CSTR. Cross-sectional diagram depicting the experimental arrangement of the CSTR used.

When this reaction system is operated in a continuous stirred tank reactor (CSTR) (Fig. 2, Supplementary Figs 1–3, a vessel to which the individual reactants and mixed products are flowing in and out continuously) this runaway reaction can be suppressed by the continuous flushing of the catalytic products from the vessel, trapping the reaction in a state of high pH (low acid) kinetic equilibrium. However, disturbing this process by the light-generated addition of a small amount of acid causes the system to quickly move to the low pH (high acid) thermodynamic equilibrium state. The acidic solution then actuates (contracts) a polyacrylic acid (PAA) based pH-responsive hydrogel (a tough semi-interpenetrating polymer network of PAA and an ether-based polyurethane, water-equilibrated Young’s modulus 204.5 ± 77.2 kPa, see Supplementary Information, Section S6 for complete mechanical characterisation).

Such combinations of non-linear chemical reactions and responsive hydrogels have previously been used to induce autonomous mechanical oscillations, as first reported by Yoshida *et al*.^26^. Although most subsequent research in this area has employed the Belousov−Zhabotinsky (BZ) reaction^27^, there has been use of other systems including work by Horváth with the Landolt system^28^. Here we harness excitability^29, 30^, another property of such autocatalytic reactions but one which has not been applied to the actuation of hydrogels. While light has been used both to modulate oscillations from nonlinear reactions in the past^31, 32^, and as the sole source of pH change for hydrogel actuation^33–35^, the opportunity to apply light merely as the low energy trigger for an excitation and resulting high energy pH-induced actuation has not yet been reported.

## Results

**Figure 3:**
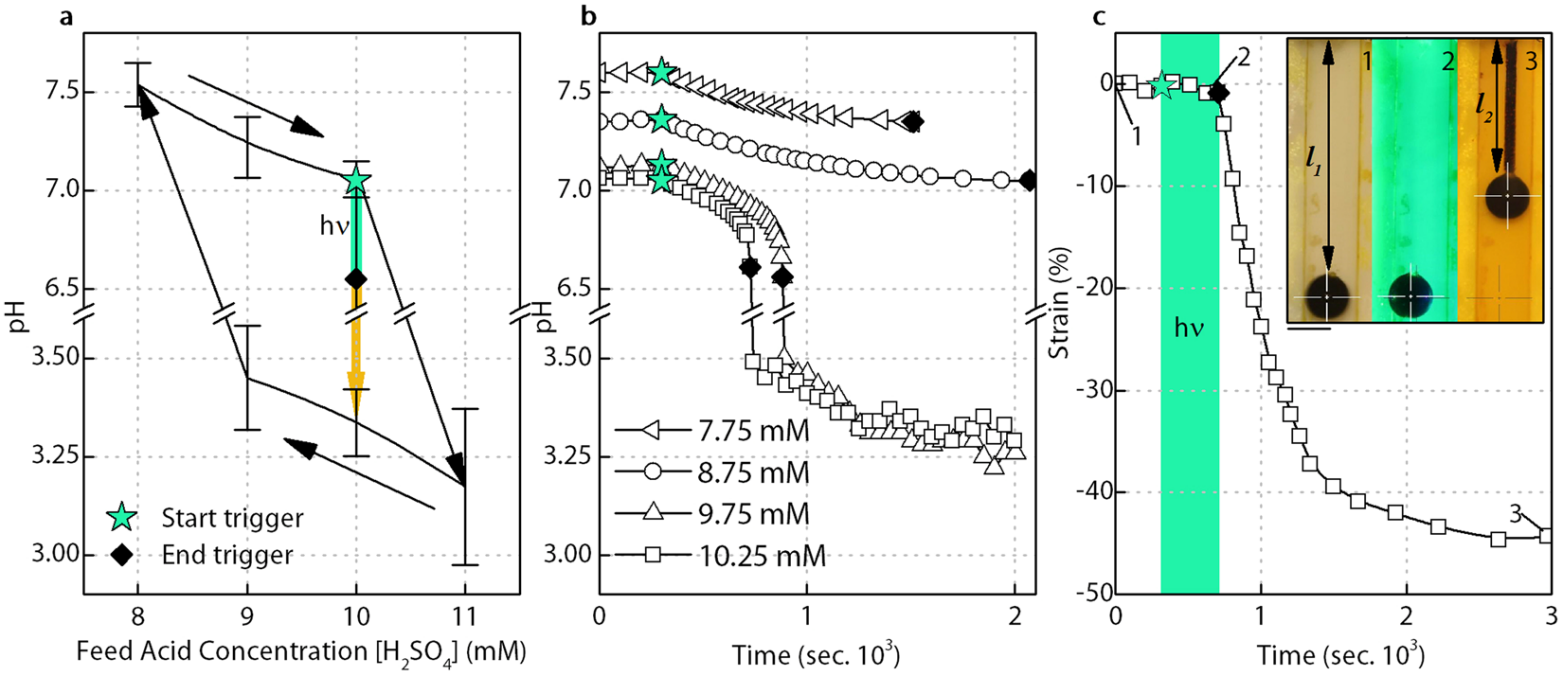
Bistable system response, critical feed acid concentration for successful light-triggering and resulting actuation strain. (**a**) The observed bistability between the high pH kinetic equilibrium and low pH thermodynamic equilibrium states. Error bars ± 1 standard deviation. Illumination near the end of the high pH branch of bistability triggers a jump to the low pH state where actuation occurs. (**b**) Successful triggering only occurs above a critical feed acid concentration (9.5 mM – 9.75 mM), as shown for illumination of systems with feed acid concentrations ranging from 7.75 mM to 10.25 mM. (**c**) The resulting actuation strain response (44.7%), calculated as the change in hydrogel length normalized to the unstrained, swollen hydrogel length (*l*_1_ *−l*_2_ *)/l*_1_ × 100, is comparable with that of biological muscle. Photo inset of artificial muscle actuation states, black sphere adhered to hydrogel and used for tracking (1) prior to illumination, the hydrogel of extended length *l*_1_ is completely transparent (2) at the end of illumination the hydrogel still has not contracted (3) at the end of actuation it has now actuated to length *l*_2_. Scale bar 5 mm. The clear swollen hydrogel only becomes visible through the accumulation of iodine after triggering. All results with 12 mm long, 1 mm diameter (unswollen dimensions) micro-porous hydrogel in place (actuation blocking stress of approximately 23 kPa).

In this work, the iodate concentration (19 mM) and sulphite concentration (60 mM) are kept constant and at a ratio of 3.16, close to the stoichiometric composition of Reaction 2 and 3 where the greatest pH drop can occur^28^ (Supplementary Fig. 4). Here the system is operated with a slight excess of sulphite to avoid a situation where excess iodate can react with iodine in the low pH state to consume protons and raise the pH again^36, 37^. The CSTR was also fed with thiosulfate (6 mM), used to reduce the range of feed acid concentrations over which the system is bistable (Fig. 3a, Supplementary Fig. 6)^38–40^. The PAG is added to all feed solutions at a concentration of 10 mM, close to its solubility limit, ensuring the maximum concentration of PAG is present in the CSTR and that the greatest photo-active response is possible. Feed concentrations for the catalytic component, sulfuric acid, is then varied whilst keeping the total flow rate constant (0.083 ml s^−1^) and the resulting system response observed.

As shown in Fig. 3a, the system is bistable with respect to the feed acid concentration. While the system remains indefinitely in the high pH state whilst being fed with fresh reactants, illumination will trigger the system to the low pH state if the feed acid concentration is sufficiently high, as demonstrated in Fig. 3b. Above this critical feed acid concentration (9.5 mM–9.75 mM) the activity of the PAG shifts the system from the high pH kinetic equilibrium to a point where the acid autocatalysis will force the system to the low pH thermodynamic equilibrium state, despite the initial light trigger being removed. The actuation strain response is shown in Fig. 3c, with 45% strain – comparable with that of biological muscle – being recorded. The jump to the low pH state is accompanied by a change in solution colour from clear to the yellow of iodine, with actuation strain recorded by the movement of a black polymer sphere adhered to the end of the hydrogel (Fig. 3c photo inset, Supplementary Movie 1).

**Figure 4:**
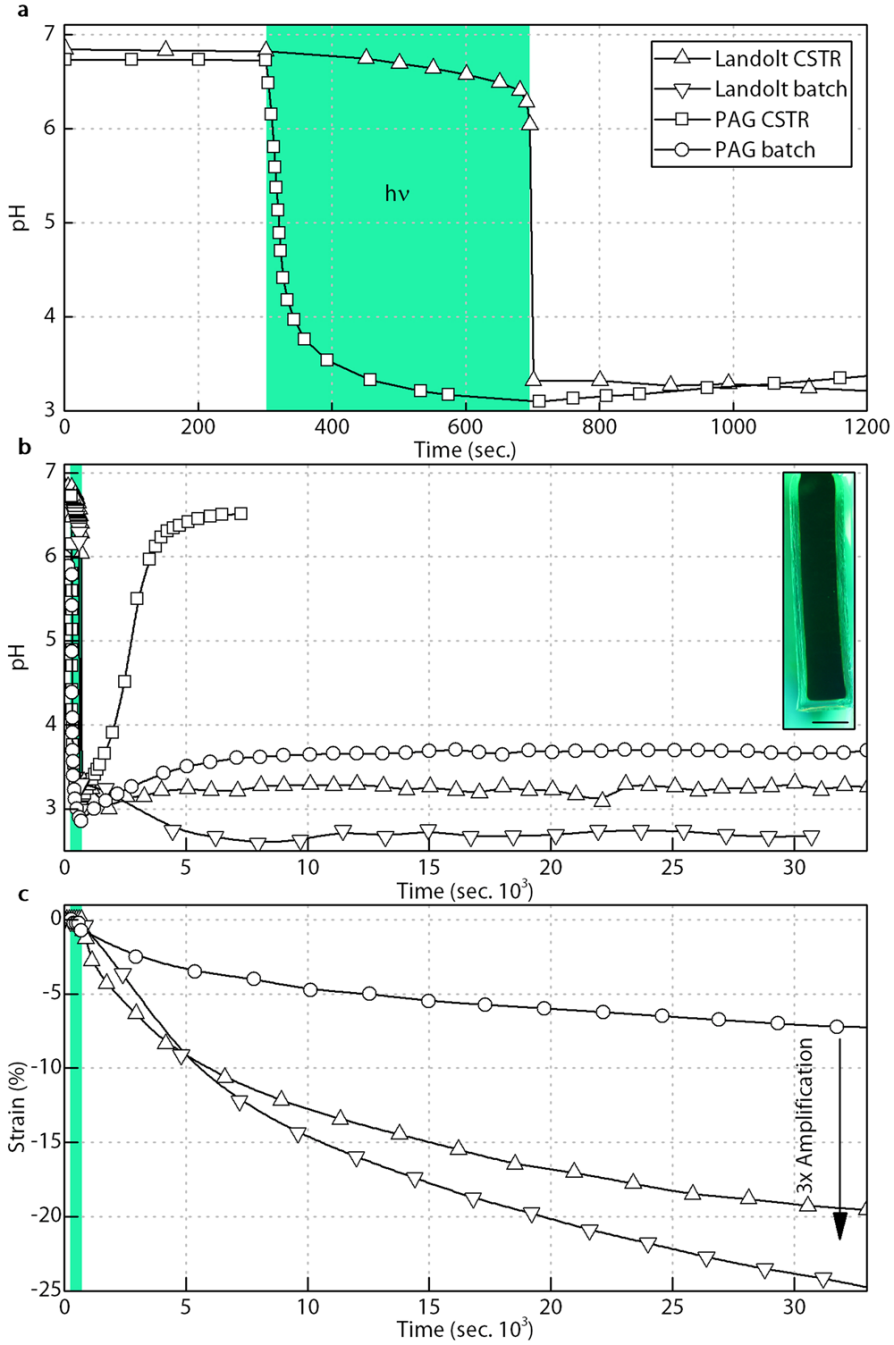
Amplified system response. (**a**) The Landolt CSTR illumination response shows a comparable, albeit delayed pH change to that offered by the PAG alone. (**b**) The use of a large enough hydrogel combined with post illumination PAG and Landolt batch operation, where the CSTR pumps are turned off, reveals the role of pH buffering. (**c**) The resulting three-fold actuation strain when comparing the PAG batch result (no diffusion rate restrictions) to either the Landolt system under CSTR or batch operation (Supplementary Movie 2). The use of larger hydrogels does result in part of the hydrogel’s interior being trapped in the low pH state, as shown by the dark region in the photo inset of the hydrogel artificial muscle, likely limiting the resulting actuation and indicating that further refinement of the system could yield even larger amplification values. Here we no longer employ the black tracking sphere as per the inset of Fig. 3, as the edges of the non-porous gel used remain visible in all states. Scale bar 5 mm.

While this behaviour successfully demonstrates the separation of control and actuation energy, this approach only has utility if, like in our biological muscle systems, the control signal is amplified, with the supply of actuation energy being greater than that of the trigger energy. Interrogation of Reaction 2 and 3 suggests a substantial chemical amplification of the light generated acid (control signal) should be achieved by a successful trigger event. However, as shown by the pH results in Fig. 4a for a CSTR illuminated with solutions composed of both our Landolt system (10.25 mM feed acid) and a system composed of only the PAG (with added sodium chloride, 161.25 mM, to approximate the Landolt system ionic strength), this amplification does not display itself as an increased reduction in pH. That is to say, after illumination and successful triggering to the low pH state, the free acid concentration of the Landolt system is comparable to that generated by the PAG alone. This apparent lack of amplification results from the buffering influence of the sulphate generated (${{\rm{HSO}}}_{4}^{-}\rightleftharpoons {{\rm{H}}}^{+}+{{\rm{SO}}}_{4}^{2-}{{\rm{pK}}}_{{\rm{a}}}\approx 2$) ^41, 42^. Therefore, to demonstrate the real amplification potential, the pH-responsive hydrogel used must be sufficiently large so as to have a high global PAA concentration that will consume meaningful amounts of acid from solution. If there is a relatively high global concentration of PAA then the pH drop from the comparably low concentration PAG will be buffered by the hydrogel PAA protonation ($-\mathrm{COOH}\rightleftharpoons {{\rm{H}}}^{+}+-{{\rm{COO}}}^{-}{{\rm{pK}}}_{{\rm{a}}}\approx 4.2$), thereby reducing the available H^+^ concentration and suppressing further actuation. However, in the case of the Landolt system the influence of hydrogel protonation will be buffered by the even higher concentration of bisulphate present (HSO_4_^−^), maintaining a low pH and a higher level of actuation than that generated by the PAG alone.

This amplification is shown more clearly by changing the pH-responsive hydrogel from a 12 mm long, 1 mm diameter porous sample as used in Fig. 3, to three 20 mm long, 5 mm diameter non-porous hydrogels (actuation blocking stress of approximately 287 kPa). With this significant increase in total hydrogel volume, the ratio of unswollen hydrogel volume to CSTR volume is around 1:60, giving a global PAA concentration of approximately 6 mM. The resulting pH responses are shown in Fig. 4b for the PAG and Landolt systems under both normal CSTR operation and for cases where the CSTR pumps are stopped after illumination or triggering to the low pH state, referred to as batch condition experiments. Batch operation such as this allows for the system behaviour to be observed in isolation of restrictions caused by the hydrogel actuation rate. As expected, both the Landolt CSTR and batch systems remain at low pH, while the pH of the PAG batch system rises back towards the hydrogel pK_a_.

The photo-triggering and strain amplification of the larger hydrogel system is shown in Fig. 4c (Supplementary Movie 2). Without this increase in hydrogel volume the recorded strain amplification is negligible (Supplementary Fig. 5). As per Fig. 4b, the Landolt system strain is compared to that generated by the photoacid in batch operation (CSTR pumps turned off after illumination) to avoid any hydrogel diffusion rate restrictions, and thus record the true amplification generated. It can be seen that when comparing the PAG strain result to either the Landolt in CSTR or batch operation (pumps off one minute after trigger) a near three-fold amplification is observed (from 7% for PAG batch to 20% for Landolt CSTR or 25% for Landolt batch).

Strains from triggering the Landolt system as high as 45% have been recorded (Fig. 3c, Supplementary Movie 1), which would yield a six-fold amplification if repeated with the larger non-porous hydrogels. It is thought that the strain in this experiment is being hindered by the Landolt system moving to the low pH equilibrium state in the hydrogel core as a result of diffusion rate limitations. This is indicated by the dark hydrogel interior visible in the photo inset of Fig. 4. Continued increases in the global concentration of PAA in the system may also yield increased amplification. Although this benefit could occur, consideration also needs to be given to the relative rate of hydrogel protonation to ensure the PAG trigger is not excessively buffered by the hydrogel prior to triggering, inhibiting the jump to the low pH state.

## Discussion

This work demonstrates the principle of using light-triggered acid autocatalysis for the molecular-level amplification of control signals and resulting actuation of soft artificial muscles. While scope exists to fine-tune this system and explore other combinations of acid autocatalytic reactions and pH-responsive hydrogels, improving speed, amplification and working towards such properties as passive reversibility (see Supplementary information), it is the intention to take the general principle of photo-triggered autocatalytic systems demonstrated herein and apply it to inherently more robust and powerful artificial muscle materials. While the pH-responsive hydrogel employed represented a facile method to investigate the use of photolatent acid autocatalysis to control and power artificial muscles, the hydrogel speed of response and fragility (hydrogel samples would tend to fail mechanically after around three months of testing) hinders the practical implementation of the concepts investigated. As such, the transfer of the autocatalytic approach to different artificial muscle systems is a key consideration for the application of the findings presented, with preliminary work to this regard involving pneumatic systems driven by gas evolution reactions currently being investigated.

Furthermore, global system efficiencies need to be considered in future work. While this study has demonstrated control signal amplification in terms of mechanical outputs, the photo to mechanical energy amplification has not been quantified. In addition, a number of practical engineering aspects will need to be considered in moving from the experimental setup (Fig. 2), to applied actuator configurations, aspects such as the development of soft and compact CSTR vessels and their design integration with both artificial muscles and light sources. Improvement of the underlying chemistry is also necessary to facilitate triggering from the small light inputs that can be delivered via fibre optics, and for competitive energy storage densities to be achieved. Beyond these challenges the unique nature of the wet, vascular system proposed is expected to yield beneficial new avenues for investigation, for example the addition of functionalities such as sensory or self-healing capabilities.

While this work is a first step in the development of complementary biomimetic control and energy supply systems for artificial muscle materials, great potential has been shown for application of photo-triggered autocatalysis. Moreover, the molecular-level integration of control signal amplification, such as that demonstrated here, will be a key requirement for both the realization of highly functional actuation systems, and the successful exploitation of soft artificial muscle technology.

## Methods

Unless otherwise stated, reagent grade chemicals were purchased from Sigma Aldrich (Gillingham, UK) and used as received.

### pH-responsive Hydrogel

The pH-responsive hydrogels employed were tough semi-interpenetrating polymer networks of polyacrylic acid (PAA) and an ether-based hydrophilic polyurethane (PU), similar to those prepared by Naficy *et al*.^43, 44^. PU (Hydromed D3, AdvanSource Biomaterials, Wilmington, USA) was dissolved in a mixture of ethanol (EtOH) and deionised (DI) water (Purelab Prima DV 35, ELGA, High Wycombe, UK) 95:5 (v/v) at a ratio 1:10 (w/v). Acrylic acid (AA, 1:5 (v/v), based on PU solution), N,N′-methylenebisacrylamide cross-linker (1.1 mol% based on AA) and α-ketoglutaric acid photo-initiator (2.1 mol% based on AA) were added to the PU stock solution to form the hydrogel pre-polymer. Porosity was achieved using a porogen leaching method, with approx. 220 μm sodium chloride crystals (Fisher, Loughborough, UK), insoluble in the EtOH-based pre-polymer, used as the template material. Hydrogels were formed by pre-polymer injection into NaCl packed glass micropipette tips (internal diameter approx. 1 mm), or empty 1 mL plastic syringes (internal diameter 5 mm) before photo-curing for 3 hours (365 nm, 4 W). After curing the hydrogels were soaked in regularly changed DI water for 2 days to both leach out the NaCl and precipitate the PU out of solution forming the second polymer. The resulting hydrogel strikes a balance between mechanical robustness and swelling performance. Unlike many tough hydrogels it does not rely on any ionic networks, which would be heavily influenced and thus be incompatible with the dynamic ionic environment of the experiments conducted. Test hydrogels were cut to length and secured to an acrylic frame and tracking sphere with adhesive (Loctite 4850) prior to testing.

### Continuous Stirred Tank Reactor (CSTR)

Five stock solutions were supplied to the CSTR (Fig. 2), all containing 0.01 M 2-nitrobenzaldehyde. Solution A (potassium iodate 0.109 M) and solution B (sodium sulphite 0.3 M) were driven by the same motor, with concentrations determined from the individual pump calibration results to keep their feed ratio fixed at 3.16. Solution C (sodium thiosulfate 0.1 M), D (sulfuric acid 0.089 M) and solution E (PAG only, used to maintain a fixed flow-rate of 0.083 ml s^−1^ despite changing acid feed concentrations), were driven by separate motors. The residence time of reactants in the 60 ml CSTR was kept constant at 720 sec. Reactants were fed by five peristaltic pumps (0.22 mL/rev, 100.PH.030/4 Williamson, Poynings, UK), individually calibrated and driven by Arduino controlled stepper motors (200 steps/rev). A rectangular glass reaction vessel with overflow spout formed the CSTR. Through the vessel’s acrylic lid three 18 G needles connected to micro-bore tubing to feed reactants to the bottom of the vessel. Solution B and D (sulphite and sulfuric acid) were mixed just prior to injection in order to limit local acidification^39^, while C and E were also premixed in this fashion. Nitrogen was continuously fed to the reactor to reduce atmospheric contamination. The vessel was vigorously stirred by a magnetic stirrer bar located near the injection points. pH and temperature were both recorded in the CSTR using an S20 SevenEasy pH meter (Mettler Toledo, Leicester, UK) with InLab Expert probe. The vessel was surrounded by a flowing water jacket, which combined with the temperature control of the hotplate kept the vessel at 25 ± 0.2 °C under normal operation and limited temperature rises during illumination and exothermic pH drops to 0.5 °C.

### Light Source

The CSTR is surrounded on three sides by mirror shielding into which are built two 10 W, 520–535 nm, 445–556 lm radiant flux LEDs (Ledengin LZ4–40G108-0000) and two 10 W 405-410 nm, 3–3.8 W radiant flux LEDs (Ledengin LZ4-40UA00-00U8), all fan cooled. These were fed at a constant 700 mA using an Arduino controlled BuckPuck (3023-D-E-700, LUXdrive, Randolph, USA).

### Imaging

Photos of the hydrogel were taken using a Canon 450D, equipped with EF 100 mm f/2.8 USM macro lens for minimal geometric distortion. Sample lighting was provided by a LED light ring with orange filter to ensure no incidental activation of the photoacid could occur. Images were post processed with Matlab’s image processing toolbox used to track the location of the black plastic sphere adhered to the bottom of the hydrogel.

### Data availability

The full set of artificial muscle images and accompanying pH and temperature data is available for download from the Research Data Repository of University of Bristol at http://dx.doi.org/10.5523/bris.u62vi54jjlo11wc6hhp4zo5nj.

## Electronic supplementary material

Supplementary Movie 1

Supplementary Movie 2

Supplementary Information
