# Supplementary material for: Light-Triggered Soft Artificial Muscles: Molecular-Level Amplification of Actuation Control Signals
Source: Sci Rep. 2017 Aug 23;7:9197. doi: 10.1038/s41598-017-08777-2 (PMC5569079; doi:10.1038/s41598-017-08777-2)
Supplement: Supplementary file 3 — Supplementary Information [file 41598_2017_8777_MOESM3_ESM.pdf]

Light-Triggered Soft Artificial Muscles:

Molecular-Level Amplification of Actuation Control Signals

SUPPLEMENTARY INFORMATION

Michael P. M. Dicker[1]*, Anna B. Baker[1,2], Robert J. Iredale[1], Sina Naficy[3,4], Ian P. Bond[1],

Charl F. J. Faul[2], Jonathan M. Rossiter[5,6], Geoffrey M. Spinks[3], Paul M. Weaver[1]

AFFILIATIONS

[1]Bristol Composites Institute (ACCIS), Queen's School of Engineering, University of Bristol, Bristol, BS8 1TR, UK.

[2]School of Chemistry, University of Bristol, Bristol, BS8 1TS, UK.

[3]Intelligent Polymer Research Institute, ARC Centre of Excellence for Electromaterials Science, University of Wollongong, Wollongong, NSW 2522, Australia.

[4]School of Chemical and Biomolecular Engineering, The University of Sydney, Sydney, NSW 2006, Australia.

[5]Department of Engineering Mathematics, Merchant Venturers School of Engineering, University of Bristol, Bristol, BS8 1UB, UK.

[6]Bristol Robotics Laboratory, Bristol, BS34 8QZ, UK.

*Correspondence to: michael.dicker@bristol.ac.uk

SUPPLEMENTARY INFORMATION

S1 EXPERIMENTAL SETUP

Supplementary Fig. 1-3 show the experimental setup used in this research and described in the methods section of the main text.

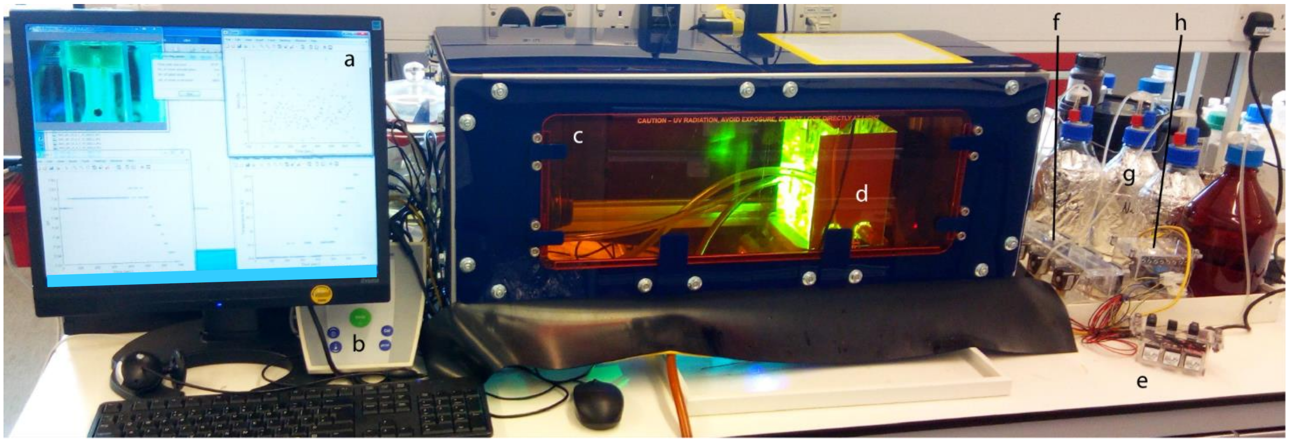

Supplementary Figure 1 | Experimental setup. (**a**) Experiment control and data acquisition. (**b**) pH meter. (**c**) Shielded box. (**d**) Reaction vessel mirror shield. (**e**) LED drivers. (**f**) Stepper motor driven pumps. (**g**) Solution tanks. (**H**) Arduino controller.

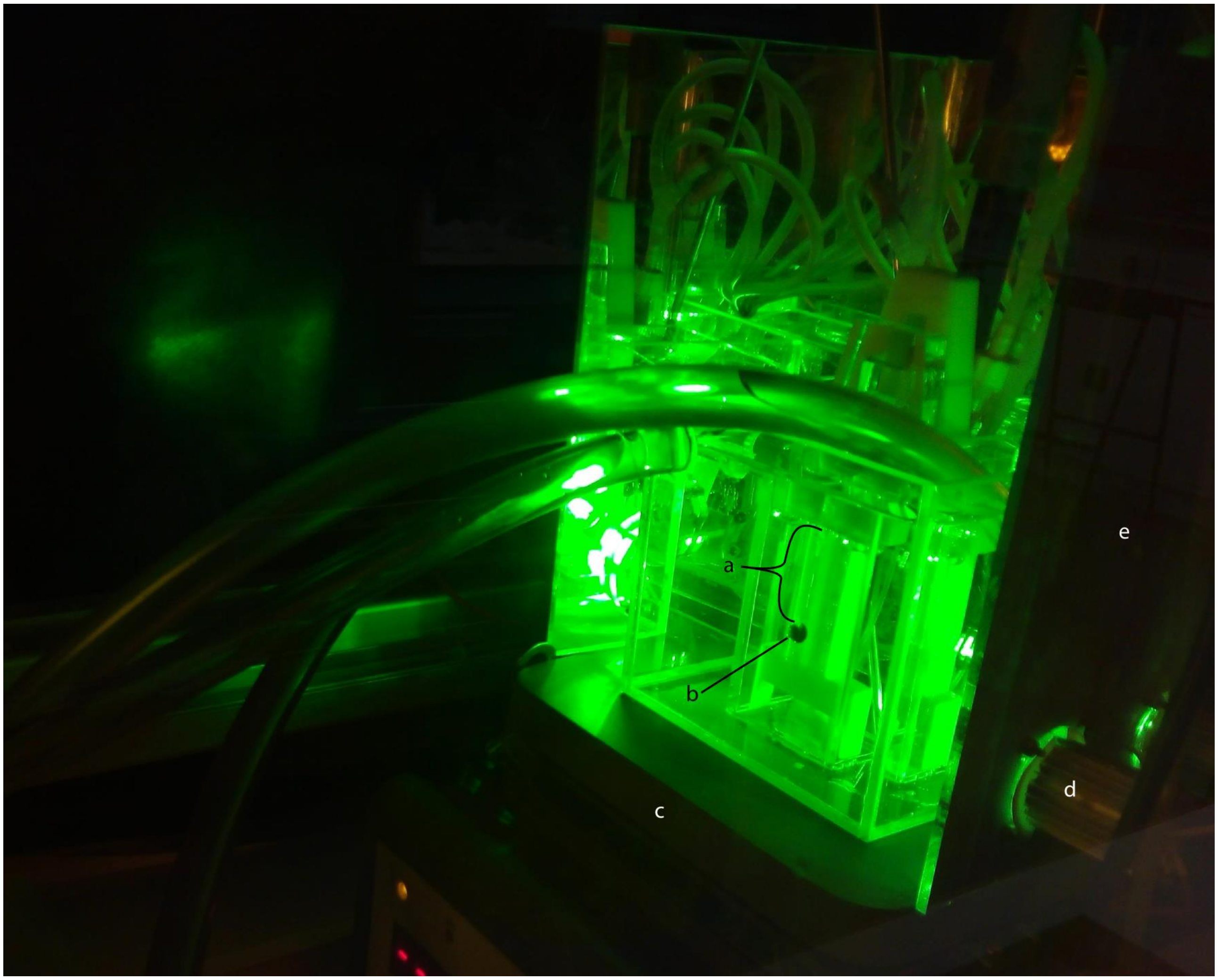

Supplementary Figure 2 | Illuminated CSTR. (**a**) Hydrogel length. (**b**) Tracking ball. (**c**) Hotplate/stirrer. (**d**) LED heatsink. (**e**) Mirror shielding.

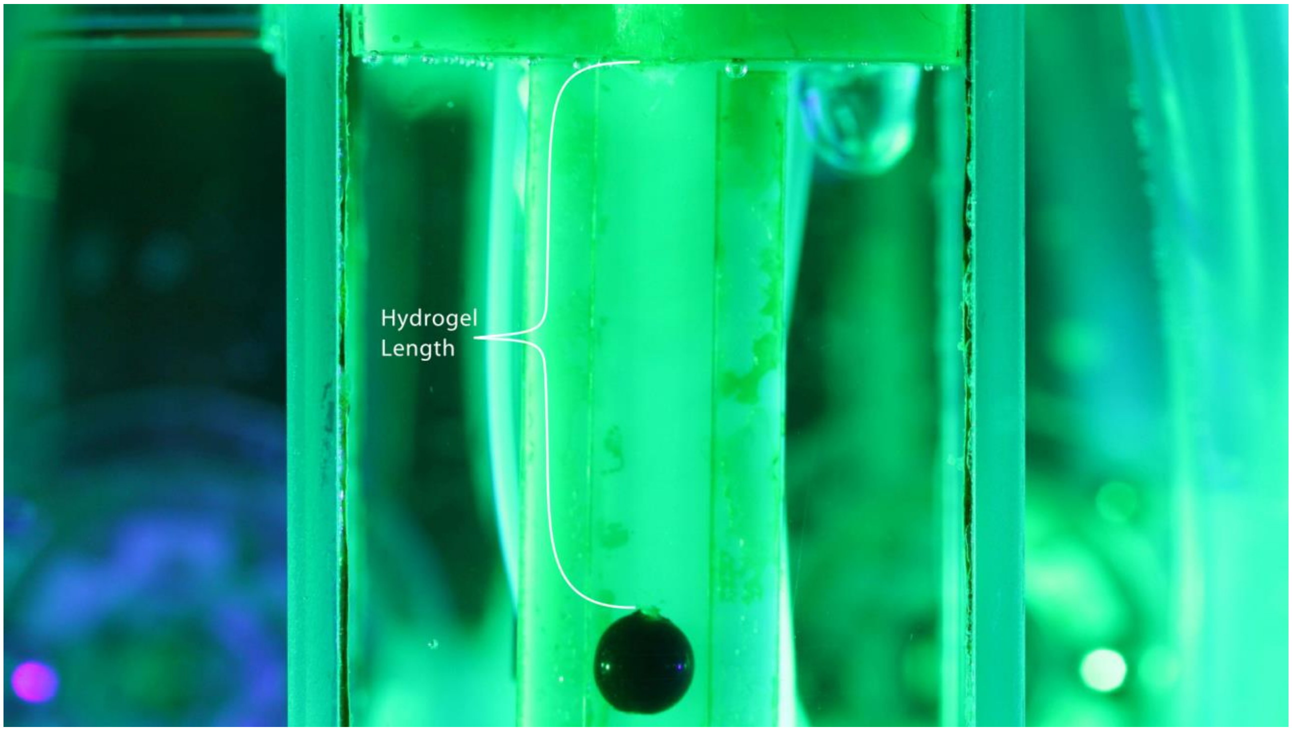

Supplementary Figure 3 | Image taken for actuation measurement.

S2 Batch Reactor Experiments

Examining the response of the Landolt system induction time (τ) in a batch reactor (closed mass flow), with reference to the starting condition, particularly acid concentration, can yield an understanding of the relationship between residence time in the CSTR and the feed acid concentration on the prevailing equilibrium state of the CSTR. The results of such a batch experiment are presented in Supplementary Fig. 4.

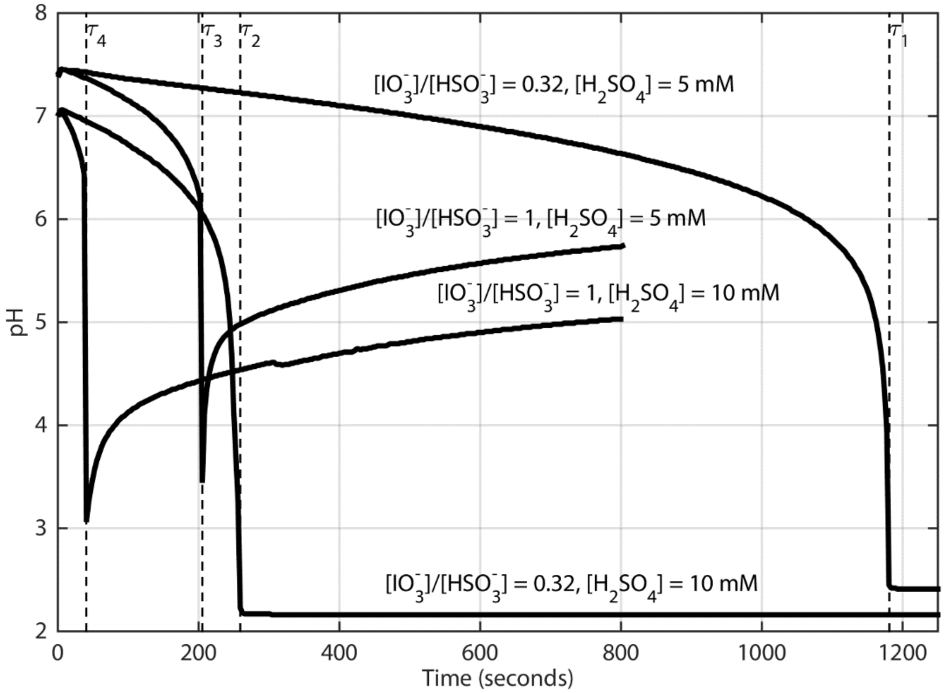

Supplementary Figure 4 | Batch experiments. Here pH is monitored from the time a solution of sulfuric acid and sodium sulphite (60 mM) is added to a small stirred vessel of potassium iodate. Both the influence acid concentration has on the time for the system to go to equilibrium (induction time τ) and the effect of the iodate/bisulphite ratio on the equilibrium pH is observed. As previously stated, the largest pH drop occurs in the vicinity of the stoichiometric ratio of Reaction 2 and 3.

S3 FURTHER PHOTO-ACTIVATION RESULTS

Supplementary Fig. 5 is another example, as per Fig. 4, of comparing the photo-triggered strain of the Landolt (10.25 mM feed acid) and PAG systems. Unlike Fig. 4, the actuation is now of the small porous hydrogel, thus the buffering influence of the hydrogel is near non-existent. As a result, we see no significant amplification in actuation.

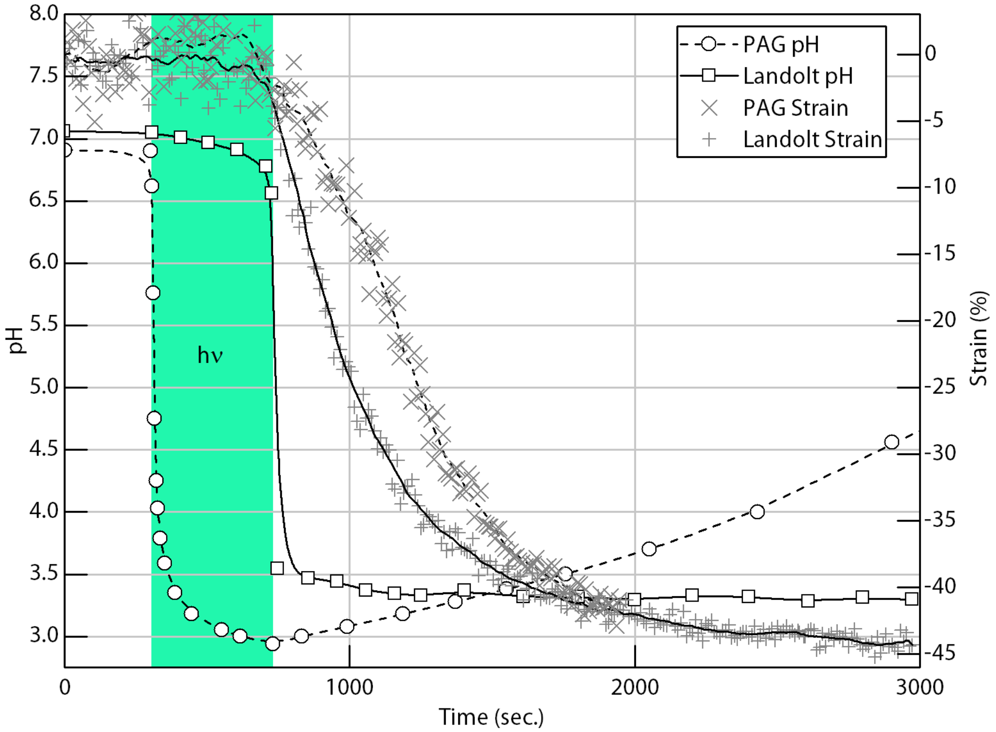

Supplementary Figure 5 | No amplification observed with small porous hydrogel. Unlike the large non-porous hydrogel, the small porous hydrogel moves around significantly in the vigorously mixed flow of the CSTR, thus the apparent noise in the strain measurement. Test at conditions 6 mM thiosulfate, 60 mM sulphite, 19 mM iodate, residence time 720 sec. Unlike Fig. 4c this is CSTR operation only, with no batch condition experiments conducted.

S4 TOWARDS REVERSIBILITY

As shown in this study, the Landolt system is characterized by large hysteresis in system pH as the concentration of acid fed to the CSTR is varied. Described another way, one could say that the pH of the reaction system is bistable, were both the high and low pH states are possible for a given range of feed acid concentrations (see Supplementary Fig. 6), with the choice of these states dependent on how the feed acid concentration is changing (its history). While this is in many regards a desirable feature for driving hydrogel actuation, locking the system in the low pH actuation state for an extended period of time, it prevents reverse actuation being achieved passively without the addition of further external stimuli. Although this study was primarily concerned with demonstrating the amplification of control signals at the molecular-level, initial investigation of the requirements for passive reversible actuation from such systems was also examined. To this end an approach investigated by Boissonade and De Kepper [1] to systematically design oscillating chemical reactions in a CSTR was employed. In reactions such as the autocatalytic Landolt operated in a CSTR the addition of increasing quantities of a component able to influence the two branches of bistability differently will see a narrowing of the bistable region to a point where it disappears. Further addition of this component, contributing more negative feedback, will begin to yield a region of autonomous oscillations (Supplementary Fig. 7) such that a cross-shaped phase diagram is revealed with respect to the bistable (feed acid) and feedback component concentrations [2]. Here the same approach was applied, but rather than aiming for the region of oscillations we instead searched for the region of minimal bistability (the cross point in the phase diagram) in

an attempt to enable the photoacid to easily jump between the high and low pH regions of the phase diagram, resulting in reversibility through operation at parameters outside the bistability band. Such an additional component able to provide negative feedback to the Landolt reaction is thiosulfate ($S_2O_3^-$) [3,4], with its influence on the system described qualitatively by Scheme S1 [5].

$$I_2 + 2\,S_2O_3^{2-} \longrightarrow 2\,I^- + S_4O_6^{2-}$$

$$IO_3^- + 6\,S_2O_3^{2-} + 6\,H^+ \longrightarrow I^- + 3\,S_4O_6^{2-} + 3\,H_2O \qquad \text{(S1)}$$

$$2\,IO_3^- + 3\,S_2O_3^{2-} + 3\,H_2O \longrightarrow 2\,I^- + 6\,HSO_3^-$$

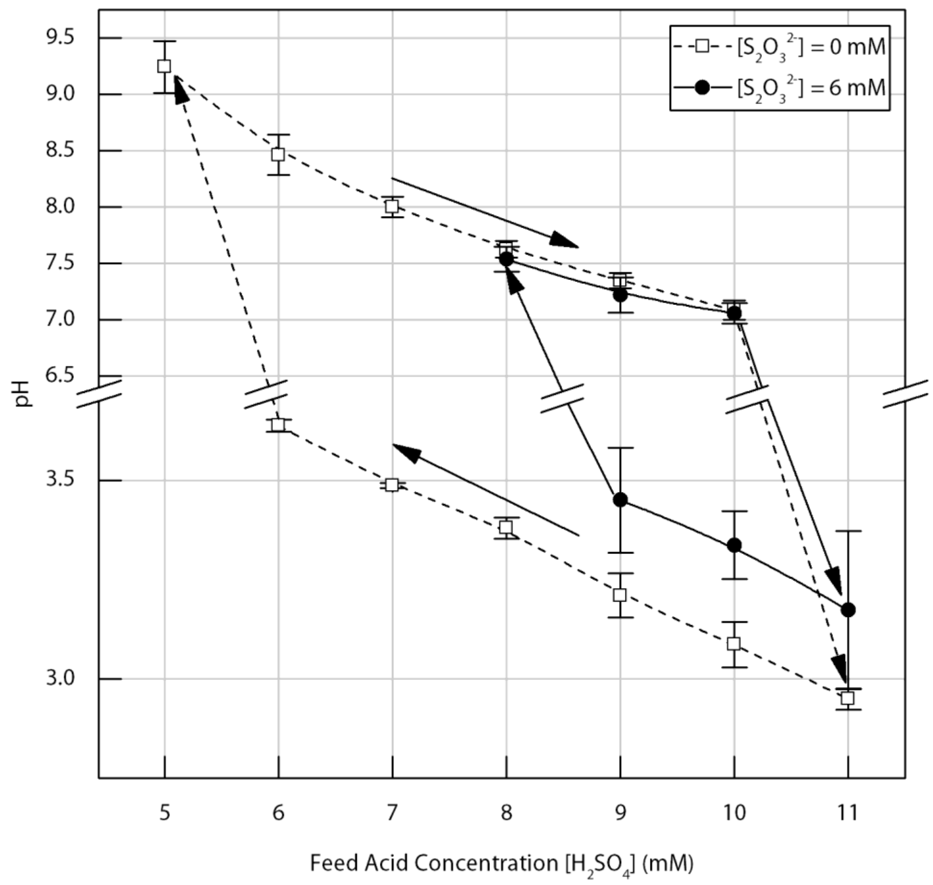

Supplementary Figure 6 | Reduction in bistability from additional of thiosulfate. The width of the bistable region was approximately halved by the addition of 6 mM sodium thiosulfate (small porous hydrogel actuator in place, 720 second residence time, 60 mM sulphite, 19 mM iodate). Error bars ± 1 standard deviation.

Supplementary Fig. 6 shows the system pH behaviour as a function of feed acid concentration when operated with both no thiosulfate feedback and thiosulfate at a feed concentration of 6 mM. While the size of the bistable region is halved through the addition of thiosulfate, it is still significant. As a result, high concentration photoacid generation is required to jump even this reduced bistability band. As shown by the illumination tests presented in Fig. 3, the PAG used in this work is, by some extent, unable to achieve this required high concentration, with feed acid concentrations very close to the end of the high pH bistability branch required before a photo-triggered jump to the low pH state is possible. While this does then result in actuation, reversibility is not achieved due to the system parameters lying within the region of bistability.

Despite much investigation, the bistable region was unable to be consistently reduced further such that reversible actuation may possibly be enabled. While system parameters that yield a region of no bistability may theoretically exist, it is likely that finding and operating in it consistently requires precise control of all system variables, beyond that which was achieved in this study.

While the level of precision of the presented experiments would seem to have been a limiting factor in the ability to reduce the bistability in the system further, it did neatly demonstrate one of the challenges of applying such complex non-linear systems to real engineering applications. For future work in such areas a greater use of modelling, combined with sensitivity analysis is expected to be hugely valuable in determining the required levels of precision, and the resulting practicality, robustness and applicability of systems prior to experimental investigation. The key findings from this initial work is the requirement for more robust feedback mechanisms in order to facilitate reversibility from these inherently bistable systems.

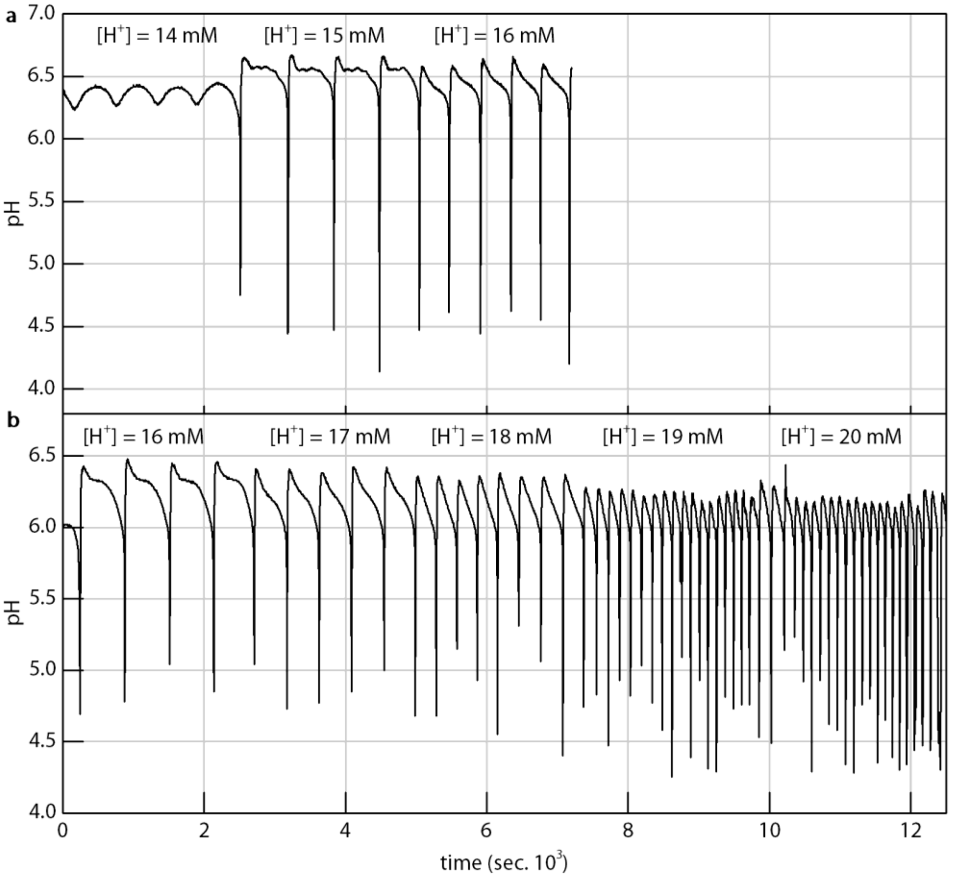

Supplementary Figure 7 | Observed oscillations. Oscillations observed at the standard test condition (small porous hydrogel actuator in place, 720 second residence time, 60 mM sulphite, 19 mM iodate). (**a**) Feed thiosulfate 10 mM. (**b**) Feed thiosulfate 20 mM.

S5 Additional Photoactive Components

In this work we examined the use of one reversible photoacid, a protonated merocyanine (MEH), and three different types of PAGs and accompanying visible light sensitizers for application as the actuation trigger: a diphenyliodonium salt (DPI) sensitized with camphorquinone (CQ), a triphenylsulfonium salt (TPS) sensitized with benzophenothiazine (BPT) and nitrobenzaldehyde (NBA).

Merocyanine

Investigations into amplifying photo-generated acid content were originally concerned with application to a reversible, yet long lived, photoacid developed by Shi *et al.*, a protonated merocyanine (MEH) [6]. While MEH produces a significant pH drop from 5.6 to 3.8 when exposed to visible light (419 nm and 570 nm absorption

peaks), the water solubility of the system is low, around 0.2 mM, thus the pH change and its actuation potential is buffered by all but the smallest pH-responsive hydrogels [7].

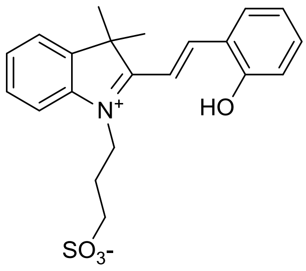

(MEH)

It was found early on in this investigation that the photo response of MEH was inhibited by the Landolt system. Specifically, a colour change similar to that observed during photo-activation of MEH occurred on contact with small concentrations of the sulfite reducing agent. Although it was envisaged that the reversibility of MEH may yield interesting phenomena, the open nature of the CSTR system employed meant that reversibility was not a necessary property of the photoacid, leading to the use of commercially available non-reversible photoacid generators in subsequent investigations.

Diphenyliodonium

Diphenyliodonium or diaryliodonium (DPI) salts, were developed by Crivello and Lam [8] who found them to be efficient cationic photoinitiators when they possessed complex metal halide anions [9]. In our work we used bis(4-tert-butylphenyl)iodonium p-toluenesulfonate, which produces *p*-toluenesulfonic super-acid upon illumination. The identity of the counter ion (MX_n^-) influences both the solubility and the strength of the acid produced. There are three challenges to the use of DPI in this work. Firstly, although it is commercially available the grades that produce the strongest acids (those with complex anions) are only readily available in pure grades for electronics manufacturing and thus are rather expensive (around $100/mmol). They also have low water solubility of 1 - 2 mM, although solubility near 80 mM is possible for diphenyliodonium chloride [10]. Finally, the absorption maximum is in the deep UV region (210 - 250 nm) [9], incompatible with our near-UV/blue/green light source, thus the addition of a photosensitizer was required to get a response from the DPI in our lighting rig. For this, Camphorquinone (CQ), a yellow dye that can sensitize the photoacid generation of DPI to blue light (470 nm absorption peak) [11–14] was used.

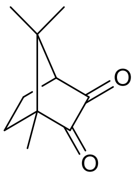

(CQ)

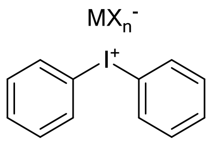

(DPI)

The challenge to also consider was the solubility of the sensitizer CQ, which is also very low in water [15]. Ultimately an aqueous system with 3 mM CQ and 1.5 mM DPI was investigated, but the acid generation was very modest, at fractions of mM quantity. However, the greatest hindrance to the use of DPI was an unforeseen interaction with the iodide (I^-) intermediary in the Landolt reaction. Upon contact with iodide the DPI forms a precipitate.

Triphenylsulfonium

Triphenylsulfonium or triarylsulfonium salts (TPS) were also developed by Crivello and Lam [16]. TPS possess the same disadvantages as DPI in terms of cost, solubility and deep UV sensitivity, but it has the advantage that it was found not to complex with any components in the Landolt thiosulfate system. However, TPS is

harder to sensitize for longer wavelength exposure than DPI, due to higher reduction potentials [17,18]. As such, it has been reported that CQ is not an effective photosensitiver for TPS [14]. Instead, benzophenothiazines (BPT) have been shown to sensitize TSS; as a result the use of a BPT (12H-benzo[b]phenothiazine prepared as per literature) was investigated [18].

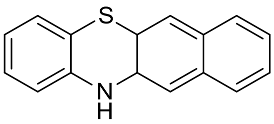

(BPT)

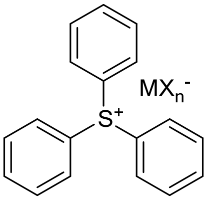

(DPI)

Unfortunately the BPT exhibited extremely poor solubility (<< 0.1 mM), both in water and 30% (v/v) solutions of DMSO. Despite this 3 mM dispersions of BPT with 1.5 mM triphenylsulfonium chloride did show a small photoacid generation that was not observed without the BPT. Despite literature to the contrary [14] CQ was successfully tested as a TPS photosensitizer (3 mM CQ, 1.5 mM TPS), displaying similar results to the DPI-CQ system.

Nitrobenzaldehyde

Ciamician and Silber were the first to demonstrate the photoacid generating capacity of 2-nitrobenzaldehyde (NBA), which transforms into nitrosobenzoic acid under UV light [19]. However, the exact mechanism is more complex [20,21], involving a ketene intermediate [22].

While the onium salts investigated prior, capable of producing super-acids, find commercial use in various cationic polymerization and lithographic processes, the moderate acid produced by the illumination of NBA has more or less only found use in research. Such investigations include the killing of cancer cells [23], the switching of enzyme activity [24] and as the sole source of energy for the actuation of pH responsive hydrogels [25,26]. Compared to the onium salts it is significantly cheaper, around \$ 1/mmol, has water solubility of 10 mM and, although all accounts only report activation with UV light (absorption maximum at 266 nm [21]); photo-activation with the near UV/blue/green lighting used in this work was found to occur without the use of a sensitizer.

S6 HYDROGEL MECHANICAL CHARACTERISATION

Testing Methods

Buffer Solutions

Unless otherwise stated the high and low pH states are where the hydrogel has been equilibrated in either a pH 11 sodium bicarbonate buffer or pH 3 citric acid buffer, corrected to an ionic strength of 0.1 M by the addition of sodium chloride. pH 3 buffer solution was composed of 0.0465 M citric acid and 0.035 M trisodium citrate. pH 11 buffer solution was composed of 0.025 M sodium bicarbonate and 0.0227 M sodium hydroxide.

Fracture Energy

Fracture energies were measured using the trouser tear test developed by Rivlin and Thomas [27]. The test is depicted in Supplementary Fig. 8 and involved a slit being formed in thin samples. The two legs of the sample were then pulled apart perpendicular to the plane of the sheet. The average force required to tear the slit open further was then determined (F_{av}) and with the thickness of the sample (h) the fracture energy (G_c) can be calculated as [28,29]:

$$G_c = \frac{2F_{av}}{h}$$

Tests were conducted with a 10 N load cell on a Instron 3343 (High Wycombe, UK) which pulled the legs apart at 10 mm min^{-1}. To aid testing sand paper was adhered to specimen grips, ensuring minimal force was required to secure test specimens, which reduced the occurrence of failure at the grip interfaces. Successful testing was only achieved with the low pH equilibrated samples, failure would occur during setup with high pH samples preventing a result from being obtained.

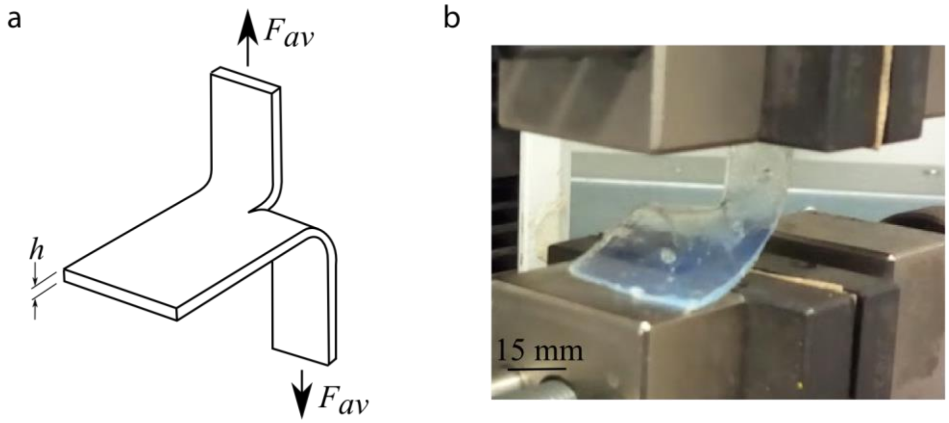

Supplementary Figure 8 | Trouser tear test for toughness. Trouser tear test for toughness evaluation and calculation of the fracture energy (**a**) schematic of test (**b**) image of test.

Tensile Mechanical Properties

Tensile tests were also conducted on rectangular strips (10 N load cell, Instron 3343, sand-paper grips, 10 mm min^{-1} travel), with the strips equilibrated at both high and low pH stretched to failure. From here the stress and strain to failure was determined as well as the work of extension, defined as the area under the stress-strain curve. The Young's modulus is calculated at 10 % strain. The gauge length was taken as the full length between grips.

Actuation Properties

Actuation was recorded in two ways. Firstly, sample weights were recorded for both pH equilibrium state (m_s) and when samples were oven dried (m_d). This yields the swelling ratio Q, defined as:

$$Q = \frac{m_s}{m_d}$$

a common measure of hydrogel swelling. Here we are more interested in the differential between the swelling ratio in the high and low states and what this means for the linear change in sample dimensions (length l), a value we refer to as strain percentage

$$\varepsilon = \frac{l_{pH\,11} - l_{pH\,3}}{l_{pH\,3}} \times 100$$

The actuation performance was also evaluated by placing loads on top of hydrogel cylinders equilibrated at pH 3 and adding the pH 11 buffer solution. Images were taken as the hydrogel swells and moves the weight, allowing for the development of an actuation force-stroke curve for the sample. Blocking stress, the normalised force generated when the sample's expansion is prevented was measured with a 10 N load cell (Instron 3433), with a similar method employed to the case of the loaded samples.

Results

Fracture Energy

The fracture energy was found to be 475.72 ± 43.03 J m^{-2} for hydrogels equilibrated at pH 3. This places their performance between that of conventional hydrogels (≈ 1 - 10 J m^{-2}) and double network hydrogels (≈ 10^3 - 10^4 J m^{-2}), representing a compromise between toughness and actuation performance. The underlying force-displacement data used to determine the fracture energy is displayed in Supplementary Fig. 9. The hydrogels had a thickness (h) of 1 mm.

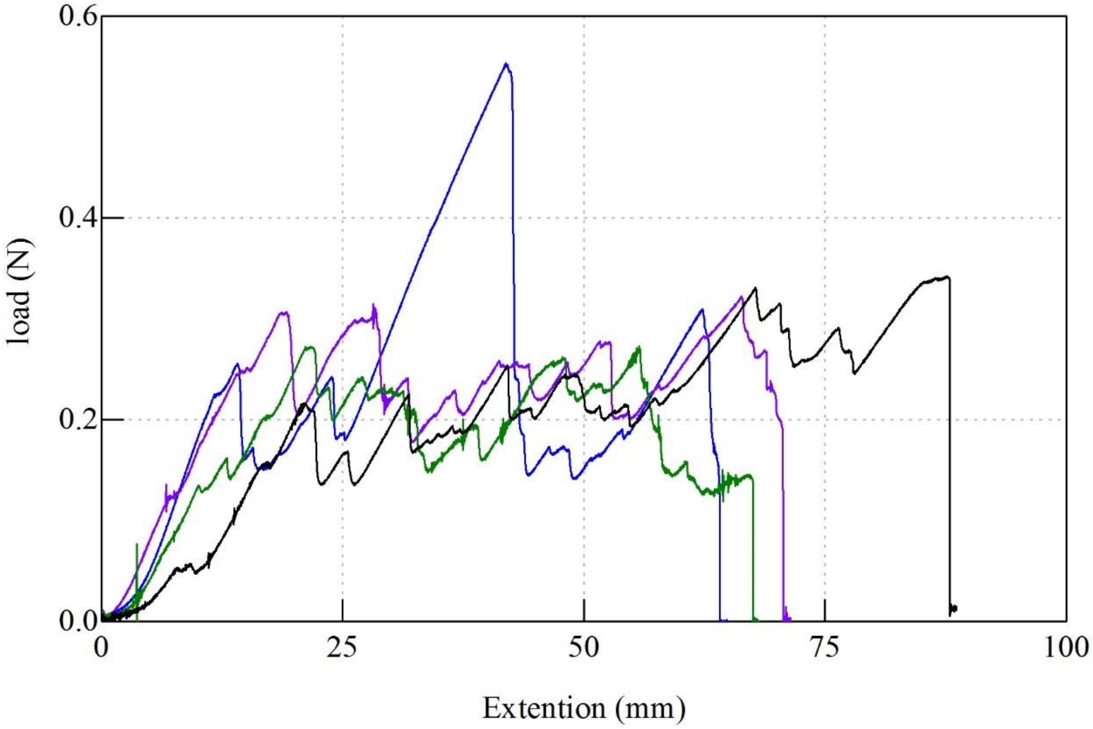

Supplementary Figure 9 | Trouser tear test output. Trouser tear test output for four samples, the loads used to calculate the average fracture force F_{av} are the repeated peaks before large falls.

Tensile Mechanical Properties

Four tensile stress-strain curves for hydrogels equilibrated at pH 3 and pH 11 are shown in Supplementary Fig. 10, with the summary of the mechanical properties included in Supplementary Table 1.

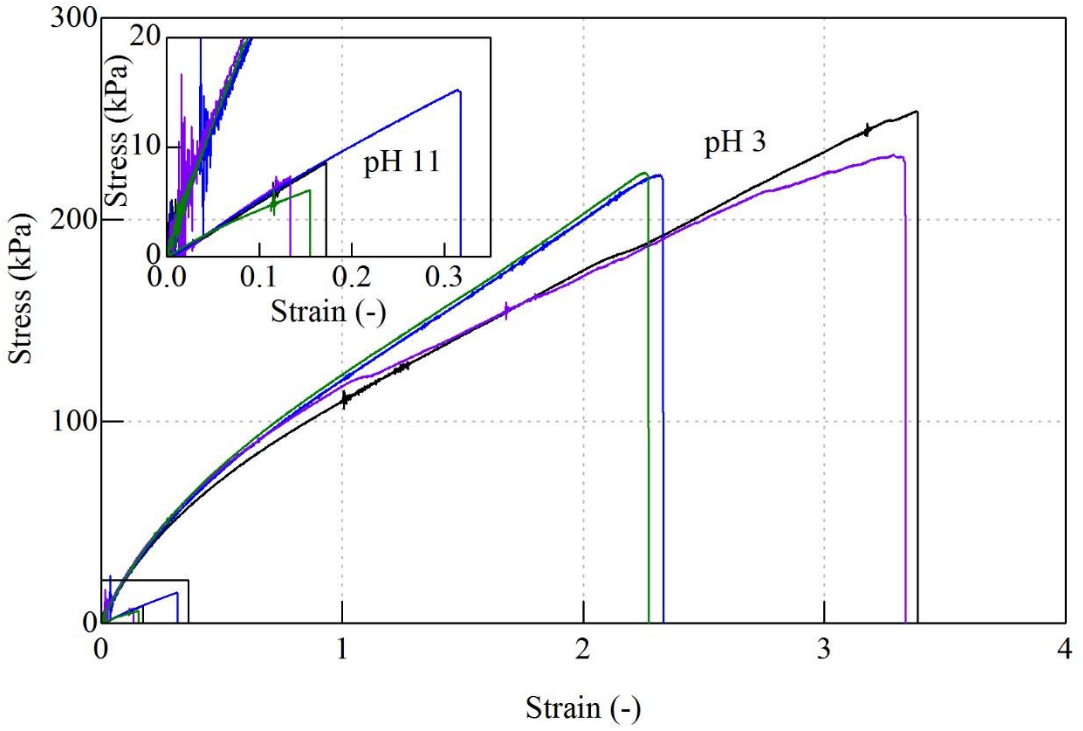

Supplementary Figure 10 | Tensile stress-strain curves. Tensile test output for four samples equilibrated at both pH 3 and pH 11. Inset showing detail to 35 % strain. The area under these curves gives the work of extension values.

Supplementary Table 1 | Mechanical performance of hydrogel.

	pH 3	pH 11
Young's Modulus (kPa)	222.38 ± 9.85	49.12 ± 4.73
Strain to Failure (%)	291.16 ± 59.80	19.47 ± 8.37
Tensile Strength (kPa)	233.04 ± 14.64	9.32 ± 4.08
Fracture Energy (J m-2)	475.72 ± 43.03	-
Work of Extension (kJ m-3)	398.46 ± 116.71	1.05 ± 0.99
Swelling Ratio (-)	2.42 ± 0.08	27.66 ± 0.58

Actuation Properties

As reported in Supplementary Table 1 the swelling ratio Q changes from 2.42 ± 0.08 to 27.66 ± 0.58 upon switching the pH from 3 to 11. The corresponding volume ratios for these samples is 2.60 ± 0.23 to 39.55 ± 1.68, with an actuation strain of 147.67 ± 20.66 %. In Supplementary Fig. 11 the actuation strain of a single sample as a function of loading, normalised to stress based on hydrogel unswollen cross-section is shown, defining the hydrogel actuator's operating window. The blocking stress from these results was found to be 287 kPa.

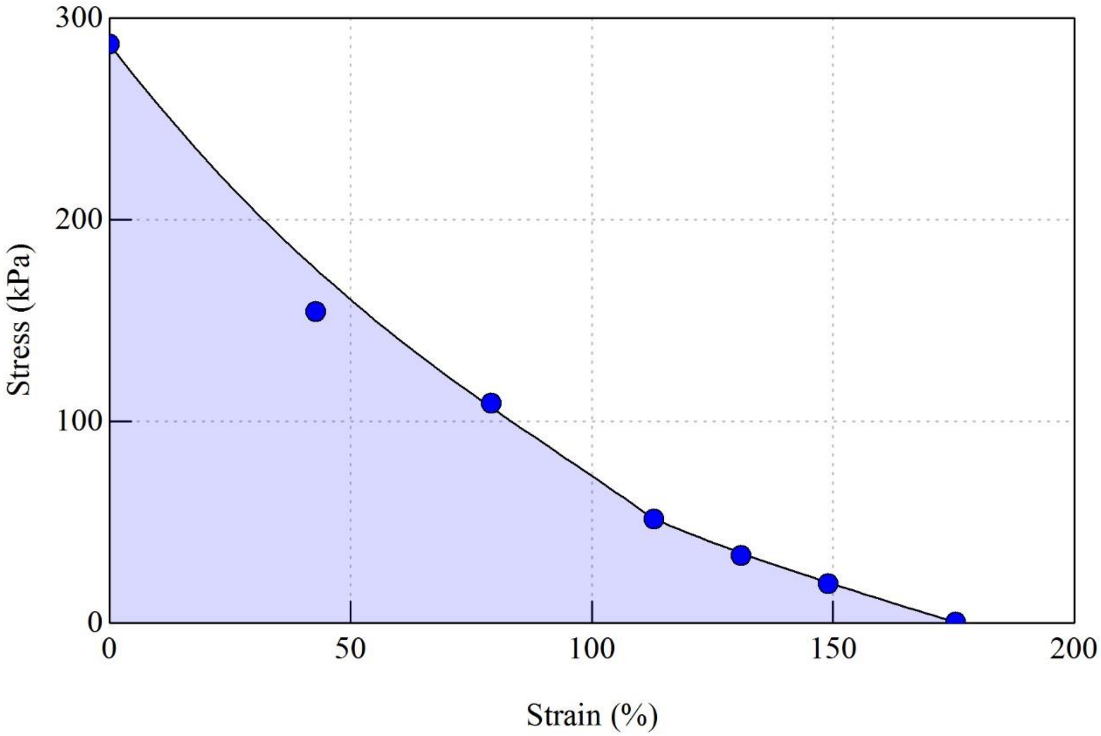

Supplementary Figure 11 | Actuation stress-strain curve. Actuation stress-strain curve, a normalised version of a force-stroke curve. Here the data points represent the bounds of actuation performance.

Supplementary Fig. 12 compares the toughness and actuation performance for the semi-IPN hydrogel used here to a number of other results from literature. Here work of extension is used as a proxy for the material toughness as fracture energy results in literature are limited, particularly for actuating hydrogels. Similarly, swelling ratio is used to asses actuating performance as it is a commonly recorded property of hydrogels. Two regions are defined is this chart for the typical performance of conventional and double network hydrogels, to which a series of connected data points are included for specific hydrogel actuators. It can be seen that the developed hydrogel's performance (d) sits in the area between these two regions, with moderate toughness and respectable actuation performance.

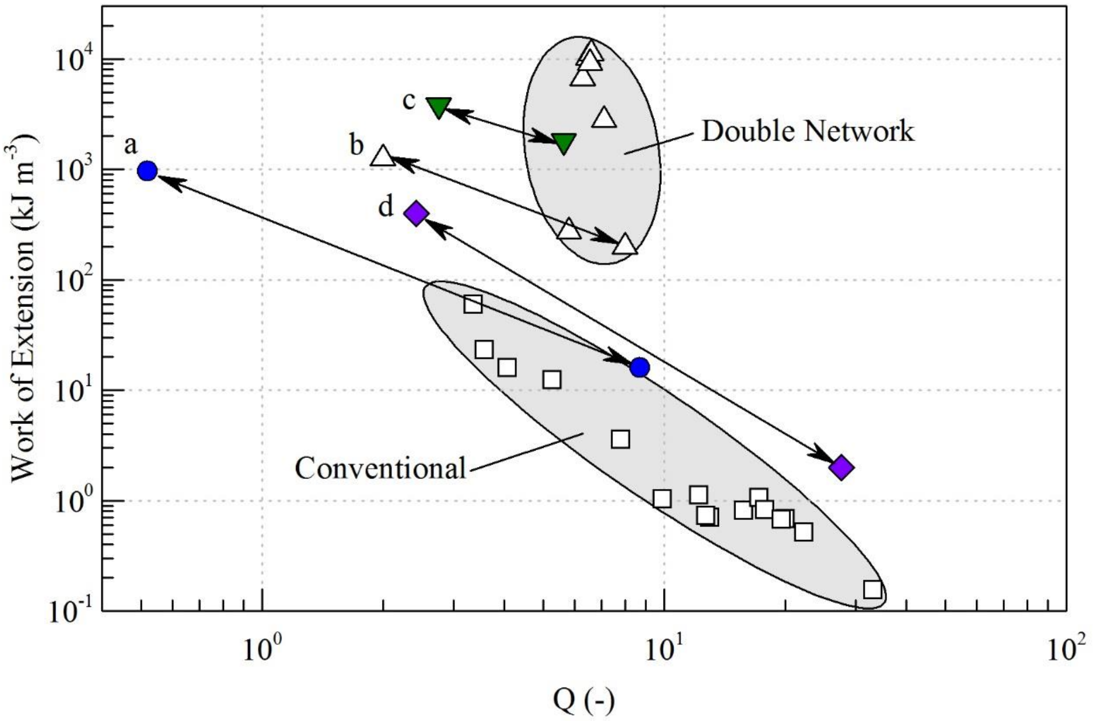

Supplementary Figure 12 | Comparisons of hydrogel work of extension and swelling ratio. Comparisons of hydrogel work of extension (representative of toughness) and swelling ratio (representative of actuation performance). Data linked by arrows are results from actuating hydrogels (**a**) Thermally-responsive ionic covalent entanglement gel of Bakarich et al. [30]. (**b**) pH-responsive double network of Naficy et al. [31]. (**c**) pH-responsive semi-IPN of Naficy et al. [32]. (d) pH-responsive hydrogel developed in this work. It can be seen from the length of the arrows that actuation performance diminishes with increasing toughness. The hydrogel developed, as with the ionic covalent entanglement hydrogel of Bakarich et al., spans the region between the weak but highly swollen conventional hydrogels and the robust but poorly swollen double network hydrogels, representing the ideal compromise of properties for actuation applications.

S7 Movies

Supplementary Movie 1 | Light-triggered muscle. Micro-porous pH-responsive hydrogel artificial muscle. Strain results shown in Fig. 3c. Feed acid concentration 10.25 mM.

Supplementary Movie 2 | Strain amplification. Comparing PAG batch and CSTR batch as per Fig. 4c.

S8 References

1. Boissonade, J. & De Kepper, P. Transitions from bistability to limit cycle oscillations. Theoretical analysis and experimental evidence in an open chemical system. *J. Phys. Chem.* **84,** 501–506 (1980).
2. Epstein, I. R., Kustin, K., De Kepper, P. & Orbán, M. Oscillating Chemical Reactions. *Sci. Am.* **248,** 112–123 (1983).
3. Rabai, G. & Beck, M. T. Exotic kinetic phenomena and their chemical explanation in the iodate-sulfite-thiosulfate system. *J. Phys. Chem.* **92,** 2804–2807 (1988).
4. Liu, H., Xie, J., Yuan, L. & Gao, Q. Temperature oscillations, complex oscillations, and elimination of extraordinary temperature sensitivity in the iodate-sulfite-thiosulfate flow system. *J. Phys. Chem. A* **113,** 11295–300 (2009).
5. Liu, H. *et al.* Pattern formation in the iodate-sulfite-thiosulfate reaction-diffusion system. *Phys. Chem. Chem. Phys.* **14,** 131–7 (2012).
6. Shi, Z., Peng, P., Strohecker, D. & Liao, Y. Long-lived photoacid based upon a photochromic reaction.

J. Am. Chem. Soc. **133,** 14699–703 (2011).
7. Dicker, M. P. M., Weaver, P. M., Rossiter, J. M., Bond, I. P. & Faul, C. F. J. Biomimetic photo-actuation: progress and challenges. in *SPIE 9797, Bioinspiration, Biomimetics, and Bioreplication* (2016). doi:10.1117/12.2219154
8. Crivello, J. V. & Lam, J. H. W. New photoinitiators for cationic polymerization. *J. Polym. Sci. Part C-Polymer Symp.* 383–395 (1976).
9. Crivello, J. V. & Lam, J. H. W. Diaryliodonium Salts. A New Class of Photoinitiators for Cationic Polymerization. *Macromolecules* **10,** 1307–1315 (1977).
10. Ichimura, K. Direct and sensitized photolysis of dispersed photoacid generators. *J. Photopolym. Sci. Technol.* **20,** 605–614 (2007).
11. Crivello, J. V. & Sangermano, M. Visible and Long-Wavelength Photoinitiated Cationic Polymerization. *J. Polym. Sci. Part A Polym. Chem.* **39,** 343–356 (2001).
12. Cook, W. D. & Chen, F. Enhanced photopolymerization of dimethacrylates with ketones, amines, and iodonium salts: The CQ system. *J. Polym. Sci. Part A Polym. Chem.* **49,** 5030–5041 (2011).
13. Schroeder, W. F., Asmussen, S. V, Sangermano, M. & Vallo, C. I. Visible light polymerization of epoxy monomers using an iodonium salt with camphorquinone/ethyl-4-dimethyl aminobenzoate. *Polym. Int.* **62,** 1368–1376 (2013).
14. Xie, X., Mistlberger, G. & Bakker, E. Visible light induced photoacid generation within plasticized PVC membranes for copper (II) ion extraction. *Sensors Actuators, B Chem.* **204,** 807–810 (2014).
15. Kamoun, E. A., Winkel, A., Eisenburger, M. & Menzel, H. Carboxylated camphorquinone as visible-light photoinitiator for biomedical application: Synthesis, characterization, and application. *Arabian Journal of Chemistry* (2014). doi:10.1016/j.arabjc.2014.03.008
16. Crivello, J. V. & Lam, J. H. W. Photoinitiated cationic polymerization with triarylsulfonium salts. *J. Polym. Sci.* **17,** 977–999 (1979).
17. Yağci, Y. & Reetz, I. Externally stimulated initiator systems for cationic polymerization. *Prog. Polym. Sci.* **23,** 1485–1538 (1998).
18. Crivello, J. V. Benzophenothiazine and benzophenoxazine photosensitizers for triarylsulfonium salt cationic photoinitiators. *J. Polym. Sci. Part A Polym. Chem.* **46,** 3820–3829 (2008).
19. Ciamician, G. & Silber, P. Chemische Lichtwirkungen. *Berichte der Dtsch. Chem. Gesellschaft* **40,** 2415–2424 (1907).
20. Abbruzzetti, S., Carcelli, M., Rogolino, D. & Viappiani, C. Deprotonation yields, pKa, and aci-nitro decay rates in some substituted o-nitrobenzaldehydes. *Photochem. Photobiol. Sci.* **2,** 796–800 (2003).
21. Donten, M. L., Hamm, P. & Vandevondele, J. A consistent picture of the proton release mechanism of o NBA in water by ultrafast spectroscopy and ab initio molecular dynamics. *J. Phys. Chem. B* **115,** 1075–1083 (2011).
22. Kuberski, S. & Gebicki, J. Evidence for a ketene intermediate in the photochemical transformation of matrix-isolated o-nitrobenzaldehyde. *J. Mol. Struct.* **275,** 105–110 (1992).
23. Kadri, N. B. *et al.* Photodynamic acidification therapy to reduce triple negative breast cancer growth in vivo. *ASCO Meet. Abstr.* **34,** e12574 (2016).
24. Kohse, S., Neubauer, A., Pazidis, A., Lochbrunner, S. & Kragl, U. Photoswitching of enzyme activity by laser-induced pH-jump. *J. Am. Chem. Soc.* **135,** 9407–11 (2013).
25. Techawanitchai, P., Ebara, M., Idota, N. & Aoyagi, T. Light-induced spatial control of pH-jump reaction at smart gel interface. *Colloids Surf. B. Biointerfaces* **99,** 53–9 (2012).
26. Techawanitchai, P. *et al.* Photo-switchable control of pH-responsive actuators via pH jump reaction. *Soft Matter* **8,** 2844 (2012).
27. Rivlin, R. S. & Thomas, A. G. Rupture of rubber. I. Characteristic energy for tearing. *J. Polym. Sci.* **10,** 291–318 (1953).
28. Muscat-Fenech, C. M. & Atkins, A. G. Elastoplastic trouser tear testing of sheet materials. *Int. J. Fract.* **67,** 69–80 (1994).
29. ASTM. *DESIGNATION: D624 - 00(2012): Standard Test Method for Tear Strength of Conventional*

Vulcanized Rubber and Thermoplastic Elastomers. (2012).
30. Bakarich, S. E., Gorkin, R., Panhuis, M. in het & Spinks, G. M. 4D printing with mechanically robust, thermally actuating hydrogels. *Macromol. Rapid Commun.* **36,** 1211–1217 (2015).
31. Naficy, S., Razal, J. M., Whitten, P. G., Wallace, G. G. & Spinks, G. M. A pH-sensitive, strong double-network hydrogel: Poly(ethylene glycol) methyl ether methacrylates-poly(acrylic acid). *J. Polym. Sci. Part B Polym. Phys.* **50,** 423–430 (2012).
32. Naficy, S., Spinks, G. M. & Wallace, G. G. Thin, Tough, pH-Sensitive Hydrogel Films with Rapid Load Recovery. *ACS Appl. Mater. Interfaces* **6,** 4109–14 (2014).
